# Quantitative magnetic resonance imaging (MRI) analysis of anterior talofibular ligament in lateral chronic ankle instability ankles pre- and postoperatively

**DOI:** 10.1186/s12891-017-1758-z

**Published:** 2017-09-12

**Authors:** Wei Liu, Hong Li, Yinghui Hua

**Affiliations:** 1 Department of Sports Medicine, Huashan Hospital, No 12, Wulumuqi Zhong Road, Shanghai, 200040 People’s Republic of China; 2 Department of Orthopedics, Taihe Hospital of Traditional Chinese Medicine, Tuanjie Xi Road No.59, Anhui, 236607 People’s Republic of China

**Keywords:** Ankle, Atfl, Instability, Repair, MRI

## Abstract

**Background:**

The aim of this study was to quantitatively evaluate and characterize the dimension and signal intensity of anterior talofibular ligament (ATFL) using 3.0 T MRI in the mechanical ankle instability group pre- and postoperatively.

**Methods:**

A total of 97 participants were recruited retrospectively in this study, including 56 with mechanical chronic ankle instability (CAI group) and 41 without ankle instability (Control group). All the subjects accepted MRI preoperatively. Among the 56 CAI patients, 25 patients, who accepted modified Broström repair of ATFL, underwent a MRI scan at follow-up. The ATFL dimension (length and width) and signal/noise ratio (SNR) were measured based on MRI images. The results of the MRI studies were then compared between groups.

**Results:**

The CAI group had a significantly higher ATFL length (*p* = 0.03) or ATFL width (*p* < 0.001) compared with the control group. The mean SNR value of the CAI group was significantly higher than that of the control group (*p* = 0.006). Furthermore, the mean SNR value of the ATFL after repair surgery (8.4 ± 2.4) was significantly lower than that of the ATFL before surgery (11.2 ± 3.4) (*p* < 0.001). However, no significant change of ATFL length or ATFL width were observed after repair surgery.

**Conclusions:**

CAI ankles had a higher ATFL length or width as well as higher signal intensity compared with stable ankles. After repair surgery, the mean SNR value of the ATFL decreased, indicating the relaxed ATFL becomes tight postoperatively.

## Background

Ankle sprain is one of the most common injuries during sports activity, with anterior talofibular ligament (ATFL) injury in most cases [1, 2]. After ATFL injury, symptomatic chronic ankle instability may develop in as many as 20% to 40% of patients even after conservative treatment, associated with a high rate of recurrence [3, 4]. To date, there are various methods to diagnose ATFL injury, including manual anterior drawer test (ADT), stress X-ray image, magnetic resonance imaging (MRI), ultrasound, arthroscopy [5]. Compared with other tools, MRI is a non-invasive powerful tool in analyzing and evaluating ATFL dimension or signal intensity [2, 6–8]. It has excellent accuracy and interobserver reliability for detecting ATFL injuries [9, 10]. However, these studies only qualitatively diagnosed ATFL with intact, or partial tear or complete tear [11].

Previously, Dimmick et al. [12] analyzed dimension and appearance of ATFL using MRI, and they found normal ATFL had mean thickness of 2.19 ± 0.6 mm and appeared homogeneously hypointense on MRI. Moreover, Delfaut et al. [13] delineated contour and signal variations of normal ATFL on MRI images, and they observed that 16 of 22 of the ATFL demonstrated a low signal intensity and 8 of 22 revealed a subtle increased signal intensity. Perrich et al. [14] also reported that intact ATFL ligament was of uniform thickness and low signal intensity and injured ATFL revealed thickened with increased internal signal intensity on MRI. However, there is lack of quantitative analysis of ATFL signal intensity in these studies.

In addition, there are various operative procedures to treat ATFL injury, including modified Broström ATFL repair and ligament reconstruction with tendon [15–18]. The modified Broström ATFL repair is the first-line safe choice for chronic ankle instability, recovering ankle stability and allowing patients return to pre-injury sport activities [15, 16]. At an average follow-up of 8.7 years after Broström repair surgery, Maffulli et al. [15] reported that ankle function improved significantly. To our knowledge, there is lack of image-based research regarding the ATFL dimension and signal intensity after ATFL surgical repair at follow-up.

Therefore, the aim of the present study was to evaluate ATFL dimension (length and width) and signal intensity on MRI in a group of lateral chronic ankle instability patients pre- and postoperatively. We had two hypotheses: (1) ATFL in CAI group had a higher signal intensity than that in ankle stable group; (2) MRI signal intensity of the ATFL decreased after surgery.

## Methods

### Participants

The study was approved by Health Sciences Institutional Review Board of our hospital, and written consent was obtained from all participants. A total of 97 consecutive participants from June 2013 to April 2016 were recruited retrospectively in this study, including 56 with mechanical lateral chronic ankle instability (CAI group) and 41 without ankle instability (Control group). All the participants had MRI scan preoperatively. CAI was diagnosed clinically by clinical history (pain or giving way, repetitive inversion sprains for more than 3 months), physical examination (ADT) and MRI. The ADT is carried out with the lower leg hanging free, knee flexed. The ankle is in 10° to 20° of plantar flexion, and the tibia stabilized with one hand and the heel grasped with the other. The heel is drawn forward with an anterior translation. It indicated the lateral ligament was injured, if the talus moved out of the ankle mortise anteriorly [19]. For the CAI group, the exclusion criteria were (1) obvious bone fracture of the ankle or foot on the affected side, (2) previous surgery of the affected limb, (3) functional ankle instability, (4) an avulsion at the fibular or talus attachment and (5) acute sprain. For the control group, ankles with healthy ATFL were recruited. Participants’ demographic data was shown in Table 1. The two groups did not differ significantly in age (*p* > 0.05) or BMI (*p* > 0.05).

**Table 1:** Participant Demographic Data of the Study Groups

	CAI group *(n* = 56)	Control group (*n* = 41)
Age, mean SD, y	30.8 ± 5.9	36.2 ± 8.9 ^n.s^
Body mass index (kg/m^2^)	25.0 ± 1.4	23.8 ± 3.8 ^n.s^
Side	Left, *n* = 32	Left, *n* = 22
	Right,*n* = 24	Right,*n* = 19
Gender, n	Males, *n* = 46	Males, *n* = 27
	Females, *n* = 10	Females, *n* = 14
Operation	AR, *n* = 22	–
	BR, *n* = 34	

*CAI* Chronic ankle instability. n.s. indicated there was no significant difference between groups

The records of 56 consecutive CAI patients who underwent ankle arthroscopy followed by repair or reconstruction were retrospectively reviewed. Patients were considered for surgery if conservative treatment failed to substantially alleviate the symptoms for at least 3 months. One senior surgeon performed all the operations as described previously [20, 21]. Under arthroscopy, an ATFL injury was diagnosed if an abnormal course of the ligament, a decrease in the tautness of the ligament, discontinuity of the ligament with or without the defect being filled by fibrous tissue, or an avulsion at the attachment to the fibula or talus [9]. Among the 56 CAI patients, 22 patients accepted ATFL reconstruction with tendons, and 34 patients accepted modified Broström repair of ATFL. Postoperatively, ankle was immobilized in a neutral position using a short leg cast. Rehabilitation exercises including isometric contraction of muscle groups around the ankle joint started from the day after surgery. The cast was removed 2-4 weeks after surgery. Among the 34 patients accepting Broström repair, 25 patients came back for follow-up investigation at approximately 6 to 12 months after surgery and underwent MRI scan.

### MRI scan and image analysis

Imaging was performed in a relaxed in a neutral position with a 3.0-T magnetic resonance imaging (MRI) scanner (MAGNETOM Verio, A Tim system, Siemens, Germany). All the participants had at least 1 h rest before MRI scan. Axial images were obtained with proton density–FS: repetition time, 3000 milliseconds; echo time, 32 milliseconds; matrix, 205 * 256; field of view, 15 * 15 cm; slice thickness, 3 mm; scan time, 1 min, 15 s. All these images were imported into Siemens Software Packages (NUMARIS/4, SyngoMR B17, Siemens, Germany) and all the calculations were made from the middle axial slices of ATFL using this software.

**Fig. 1:**
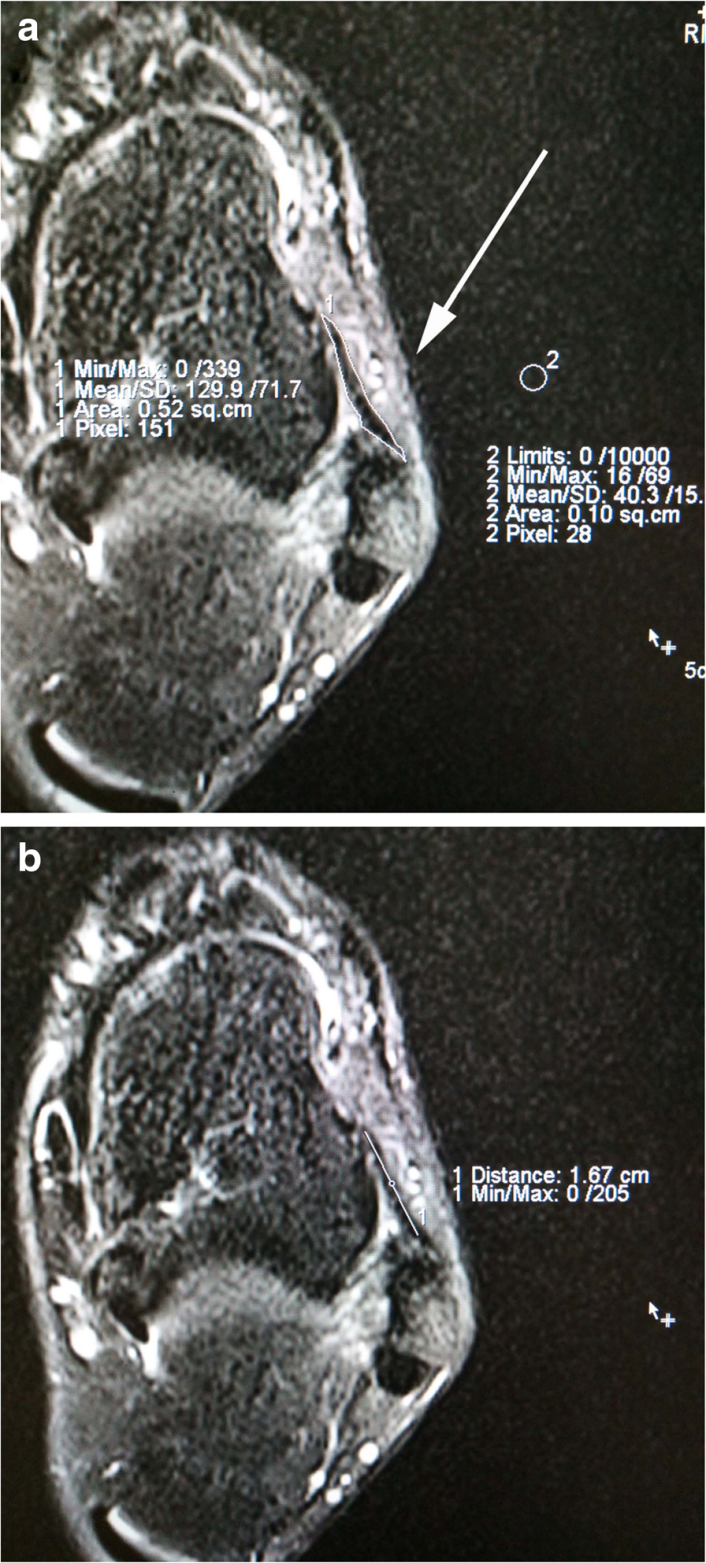
Analysis of anterior talofibular ligament (ATFL) dimension and signal intensity (SI) on axial MRI image. (1) The signal intensity (SI) was calculated at the ATFL site as well as the background site (approximately 2 cm near to the ATFL) using a region of interest (ROI). The signal/noise ratio (SNR) was calculated using the following equation: SNR = signal of ATFL / signal of background. The area of the ATFL ligament was directly measured by drawing the contour of ATFL. (2) The length of the ATFL. The ATFL was lined extending from the anterior and inferior borders of the fibula to the neck of the talus

One slice image in each ankle, which revealed the ATFL clearly, would be chosen to make MRI evaluation. The MRI evaluation focused on 3 measurements (Fig. 1): (1) The signal intensity (SI) was calculated at the ATFL site as well as the background site using a region of interest (ROI). The ROI of ATFL was defined by drawing the contour of the whole ATFL, and the signal intensity of ATFL was directly measured. The ROI of the background was defined by drawing a circle as the background site approximately 2 cm near the ATFL. To quantify the normalized signal intensity of the ATFL, the signal/noise ratio (SNR) was calculated using the following equation: SNR = signal of ATFL / signal of background [22]. (2) The length of the ATFL. The ATFL was lined extending from the anterior and inferior borders of the fibula to the neck of the talus. (3) The width of the ATFL. It was calculated the following equation: The width of ATFL = The area of ATFL / the length of the ATFL. The MRI reader was blinded to the type of operation procedure. All of the measurements were performed by two investigators and repeated measurements were made on 2 days at least 1 month apart.

### Statistical analyses

Data analysis was performed using Stata 10.0 software (Stata Corp, USA), and the data are reported as means and standard deviations. At first, post hoc power analysis was performed. If a minimal clinically important difference of 3 mm in the graft length or width between groups, it was considered a clinically significant difference of graft length or width. To quantify the proportion of the variance for all of the measurements, the intraclass correlation coefficient (ICC) was assessed by examining the interobserver and intraobserver reliabilities. The ICC was interpreted as poor if ICC < 0.4; as marginal if 0.4 ≤ ICC ≥ 0.75; and as good if ICC > 0.75. A χ2 test was used to compare the categorical variables. A two-sample t test or two-sample Wilcoxon rank-sum test was used to compare the continuous variables between groups. A paired t-test would be most appropriate when comparing the preoperative and postoperative MRI parameters of the ATFL. Spearman’s correlation coefficients were calculated between ATFL-related value (length, width or SNQ) and various factors (Gender, Age, BMI, Injury time). The significance level was set at 0.05.

## Results

For every patient, ATFL could be easily followed on axial slices from its fibular origin to its talar insertion. The ICC index of interobserver reliability was 0.79 for the graft length and 0.76 for the SNR value. The ICC index of intraobserver reliability was 0.80 for the graft length and 0.84 for the graft SNR value. The CAI group had a significantly higher ATFL length (15.9 ± 2.9 mm vs 14.7 ± 2.3 mm; *p* = 0.030) or ATFL width (3.9 ± 1.3 mm vs 2.8 ± 0.7 mm; *p* < 0.001) compared with the control group. For ligament signal, the mean SNR value of the CAI group (12.85 ± 7.80) was significantly higher than that of the control group (9.14 ± 3.58) (*p* = 0.006).

Possible associations between potential risk factors and the ATFL-related value (SNR value or length or width) were explored (Table 2). For the control group, there was no significant association between the ATFL-related value (SNR value or length or width) and gender, age or BMI. Similarly for the CAI group, there was also no significant association between the ATFL-related value (SNR value or length or width) and gender, age, BMI or injury time.

**Table 2:** Possible associations between several factors and anterior talofibular ligament (ATFL)-related value (SNR value or length or width)

Variable		Length		Width		SNR	
		rho	*P* value	rho	*P* value	rho	*P* value
Control group	Gender	−0.02	0.92	0.27	0.08	0.22	0.16
	Age	−0.02	0.92	0.11	0.48	0.30	0.06
	BMI	−0.08	0.61	0.29	0.06	0.05	0.73
CAI group	Gender	−0.20	0.14	−0.22	0.10	0.03	0.83
	Age	−0.09	0.51	−0.08	0.56	−0.01	0.94
	BMI	−0.01	0.94	0.03	0.78	−0.20	0.14
	Injury time	−0.10	0.45	0.02	0.88	−0.20	0.14

*ATFL*, *SNR* signal/noise ratio; *CAI* chronic ankle instability; *BMI* Body mass index

For the 25 patients accepting repair surgery, there was no significant difference before and after surgery regarding the ATFL length (15.9 ± 2.2 mm vs 15.5 ± 2.2 mm; *p* > 0.05) or ATFL width (4.2 ± 1.0 mm vs 4.7 ± 1.3 mm; *p* > 0.05). For ligament signal, the mean SNR value of the ATFL decreased from 11.2 ± 3.4 preoperatively to 8.4 ± 2.4 postoperatively with significant difference (*p* < 0.001).

## Discussion

In the present study, we, for the first time, analyzed and evaluated the dimension and signal intensity of the ATFL on MRI images in the chronic ankle instability group pre- and postoperatively. It was found that the CAI ankles had a higher ATFL length or width as well as higher signal intensity compared with stable ankles. In addition, the mean SNR value of the ATFL after repair surgery was significantly lower than that of the ATFL before surgery. The final results verified the two hypothesis mentioned afore.

The ATFL, a flat and quadrilateral ligament, originates from the inferior oblique segment of the anterior border of lateral malleolus, coursed anteromedially and inserted on the talar body just anterior to the lateral malleolar articular surface [23]. Previously, Taser et al. [23] directly measured the mean ATFL length and width in cadavers. They found that the mean ATFL length was 22.37 ± 2.50 mm, and the mean ATFL width was 10.77 ± 1.56 mm at proximal site, 6.75 ± 2.89 mm at middle site, 10.96 ± 2.38 mm at distal site. In 2011, Boonthathip et al. [24] measure the ATFL length and width in cadavers using MRI, and they observed that the mean ATFL length and width (the widest portion) was 21.2 ± 5.6 mm and 4.4 ± 1.0 mm respectively. Recently, Cho et al. [5] reported that ATFL length was 28 ± 3 mm in stress and 21 ± 2 mm in resting respectively using ultrasound examination. In the present study, the mean ATFL length was 14.7 ± 2.3 mm, and the mean ATFL width was 2.8 ± 0.7 mm for the stable ankles. These results were different with the analysis in cadavers or with ultrasound. This might be due to the different scan angle. MRI was performed axially, while the measurement was a long with the oblique ligament in cadavers or with ultrasound.

Previously, Dimmick et al. [12] reported the mean thickness of the intact ATFL was 2.19 ± 0.6 mm, and the mean thickness of the injured ATFL was 2.26 ± 0.53 mm in men and 2.18 ± 0.61 mm in women on MRI images. Our result regarding the ATFL width was similar with them. Furthermore, Dimmick et al. [12] investigated the mean ATFL thickness between the gender and they found no significant difference of the mean injured ATFL thickness between the men and the women. In our study, there was also no significant association between the ATFL-related value (SNR value or length or width) and gender.

In the present study, the mean ATFL length and width of the CAI group were 15.9 ± 2.9 mm and 3.9 ± 1.3 mm respectively, higher than those of the control group. Recently, Cho et al. [5] used stress ultrasound to measure the ATFL length in CAI ankles. They found that the mean value of the ATFL length in resting was 21 ± 2 mm for the CAI ankles and 21 ± 1 mm for the contralateral ankles, while there was no significant difference for relaxed ATFL length between both ankles. The difference might be due to two reasons: (1) The contralateral ankles were used as a control condition for comparisons while we used another healthy ankles as control group. (2) the CAI group by Cho et al. accepted repair procedure, while 22 patients accepted ATFL reconstruction as the reconstruction group had a higher ATFL length in the present study.

In this study, the mean SNR value of the CAI group was significantly higher than that of the control group. Generally, normal ATFL on MRI revealed a low signal intensity structures [13]. After repetitive inversion sprains, ATFL demonstrated an increased ligament signal on MRI, as fluid crossing part or all of the ligament [25, 26]. Furthermore, the mean SNR value of the ATFL after repair surgery was significantly lower than that of the ATFL before surgery. Previous investigations have reported that ligament signal intensity has a good negative linear relationship with the material biomechanical strength properties [27, 28]. Lower SNR indicated higher ligament tension. After Broström repair surgery, the relaxed ATFL became more tight and revealed a lower signal intensity. According to our results, the mean SNR value of the repair ATFL was 8.4 ± 2.4 postoperatively, which is almost the same with that of the healthy ATFL. It was presumed that the injured ATFL got back to normal after surgery. It was also believed that the SNR might be a indirect parameter to indicate if the ATFL is successfully repaired in clinical practice.

## Conclusion

Chronic instability ankles had a higher ATFL length or width as well as higher signal intensity compared with stable ankles. In addition, the mean SNR value of the ATFL decreased after repair surgery, indicating the relaxed ATFL becomes tensional postoperatively. This study provides valuable quantitative information regarding the ligament dimension and signal intensity pre- and postoperatively.

## Abbreviations

ATFL: Anterior talofibular ligament
CAI: Chronic ankle instability
SNR: Signal/noise ratio
